# Reducing Anxiety with Nature and Gardening (RANG): Evaluating the Impacts of Gardening and Outdoor Activities on Anxiety among U.S. Adults during the COVID-19 Pandemic

**DOI:** 10.3390/ijerph19095121

**Published:** 2022-04-22

**Authors:** Megan E. Gerdes, Lucy A. Aistis, Naomi A. Sachs, Marcus Williams, Jennifer D. Roberts, Rachel E. Rosenberg Goldstein

**Affiliations:** 1 Department of Epidemiology and Biostatistics, School of Public Health, University of Maryland, College Park, MD 20742, USA; gerdes.megan@gmail.com; 2 Maryland Institute for Applied Environmental Health, School of Public Health, University of Maryland, College Park, MD 20742, USA; laaistis@terpmail.umd.edu; 3 Department of Plant Science and Landscape Architecture, University of Maryland, College Park, MD 20742, USA; nsachs@umd.edu; 4 Baltimore City Extension, University of Maryland Extension, Baltimore, MD 21215, USA; mwilli65@umd.edu; 5 Department of Kinesiology, School of Public Health, University of Maryland, College Park, MD 20742, USA; jenrob@umd.edu

**Keywords:** anxiety, COVID-19, pandemic, gardening, outdoor environment, nature

## Abstract

The COVID-19 pandemic impacted mental health. Growing research has identified the mental health benefits of nature contact, including gardening. We used a cross-sectional survey to investigate the association between gardening and other outdoor activities with anxiety among U.S. adults. The RANG (Reducing Anxiety with Nature and Gardening) survey was distributed online from June–September 2020 through social media (Twitter and Facebook) and a national Master Gardeners listserv. Survey questions captured demographics, COVID-19 experiences, gardening, outdoor activities, and anxiety using the Generalized Anxiety Disorder 7-item scale. Data were analyzed using chi-square, Fisher’s exact, and Kruskal–Wallis tests, as well as logistic regression. Among participants, 46% reported anxiety symptoms. Participants who had gardened ≥ 15 years and those gardening > 8 h over two weeks had lower anxiety scores. Spending more time outdoors on weekdays also decreased anxiety scores. After adjusting for covariates, lower odds of anxiety were identified for 50–69 and 70–89-year-olds vs. 18–29-year-olds; males vs. females; and Texas vs. Maryland residents. These findings confirm increased anxiety during the COVID-19 pandemic and suggest that sustained gardening and other outdoor activities could help reduce anxiety.

## 1. Introduction

As of February 2022, there have been over 76 million cases and over 900,000 deaths caused by coronavirus disease (COVID-19) in the United States (U.S.) [1]. COVID-19 first gained attention from the World Health Organization (WHO) in late December 2019 and was characterized as a pandemic in March 2020 [2,3]. The U.S. Department of Health and Human Services (HHS) declared a nationwide public health emergency as confirmed COVID-19 cases emerged within the U.S. The Centers for Disease Control and Prevention (CDC) guidance recommended physical distancing to slow transmission [4]. Between 1 March–31 May 2020, forty-two U.S. states and territories issued mandatory stay-at-home orders with detailed directives such as “every person is ordered to stay at his or her place of residence except as necessary to perform essential activities” [5,6]. These stay-at-home orders impacted 73% of 3233 U.S. counties [5]. Increases in stress, anxiety, and depression were reported as a result of the pandemic and related fears of illness, financial insecurity/instability, and isolation [7,8,9,10]. During late June 2020, 40% of U.S. adults reported struggling with anxiety or depression, trauma or stressor-related disorder symptoms, began or increased substance use, and/or seriously considered suicide [9]. While many social and recreational activities were discouraged or prohibited during the spring and summer of 2020, individuals were still largely encouraged by the CDC and public health officials to spend time outdoors in parks and other green spaces in order to protect each other from COVID-19 transmission, but also as a way to maintain levels of physical activity [11,12,13]. Coupled together, nature exposure and physical activity have been shown to promote both physical and mental health benefits including stress reduction [11,12,13,14,15]. Furthermore, the characterization of “outdoor activity” has been eminently broad, including everything from walking in forests, exercising in urban parks, and even gardening [15,16].

There is growing evidence that interactions with nature result in physiological, psychological, and social benefits [17,18,19,20,21]. The body of research examining exposure to nature and its impact on human health has included a variety of natural settings and exposure methods [14,17,21,22,23]. Previous studies have found decreases in stress response markers, such as blood pressure and cortisol levels, after walking in nature-rich environments or even viewing nature scenes [13,21,24,25]. Psychological benefits, such as increased feelings of relaxation and happiness, increased attention restoration, reduced anxiety, and reduced mental fatigue, were found with as little as 10 min spent in a natural setting or 120 min a week in nature [11,26,27]. Dose response relationships for both the intensity and duration of green exercise (activity in the presence of nature) have also been found to have significant relationships with mental health [28].

The physical act of gardening has been shown to improve mental health by reducing depression and anxiety in adults with clinical depression and increasing emotional well-being in the general population [29,30,31]. Several international studies have identified the positive impact of gardening on mental health during the COVID-19 pandemic; however, to our knowledge, there have been no studies on the impact of gardening and outdoor activities on anxiety during the COVID-19 pandemic across multiple states in the U.S [32,33,34,35]. Through the RANG (Reducing Anxiety with Nature and Gardening) survey, we examined the impact of gardening and other outdoor activities on anxiety among gardeners in multiple states. Our primary objectives were to (1) assess the prevalence of anxiety during the summer 2020 period of the COVID-19 pandemic; (2) examine changes in gardening and outdoor practices; and (3) analyze associations between gardening and other outdoor activities with anxiety during the COVID-19 pandemic. To address these objectives, we conducted a cross-sectional study among U.S. adults using an online survey to collect information about gardening and outdoor activities during the summer of 2020 and 2019, COVID-19 experiences, and anxiety symptoms.

## 2. Materials and Methods

### 2.1. Survey Instrument

The RANG survey was developed by an interdisciplinary team of public health, landscape architecture, and Extension researchers and approved by the university’s Institutional Review Board. Questions were based on previously validated and published surveys evaluating gardening practices, outdoor activities, and the health benefits of these activities as well as validated anxiety measures (described in detail in Section 2.2) [36,37,38]. The survey consisted of 37 questions, 34 multiple-choice and 3 open-ended questions, on participant gardening habits, gardening experience, gardening duration and frequency, participation in other outdoor activities, and personal experience with the COVID-19 pandemic (Supplementary Materials). Participants were able to select more than one answer for the following questions: “What type of garden do you work in?”, “What motivated you to start gardening?”, “Our project team will be developing educational events and materials to provide information and best practices for gardening as a stress management technique. How would you like to receive this information?”, and “What information would be helpful regarding gardening?”. The RANG survey also included the 7-item self-rated Generalized Anxiety Disorder 7-Item Scale (GAD-7) to determine anxiety symptoms [39]. Psychometric evaluations of the GAD-7 found that the scale is a reliable and valid measurement tool, demonstrating sensitivity and specificity when screening for anxiety across samples with varying demographics and sizes [39,40,41,42,43]. Additionally, the GAD-7 has strong internal consistency and good procedural validity when comparing self-reported scores with scores derived from mental health professional-administered tests [39]. Finally, participants were asked to provide demographic information including their state of residence, ethnicity, race, age, gender, and education.

### 2.2. Anxiety Scores

We used responses to the GAD-7 to determine the prevalence of anxiety among participants [39]. Participants ranked how often they experienced each of seven symptoms over a two-week period (1) “not at all”, (2) “several days”, (3) “more than half the days”, or (4) “nearly every day”) (Supplementary Materials). The four possible responses to the GAD-7 questions received a point ranking of 0 (not at all), 1 (several days), 2 (more than half the days), and 3 (nearly every day). Points from individual responses to the seven questions were summed to create a continuous variable for the anxiety score. The GAD-7 was used to categorize each participant’s anxiety score as none (0–4 points), mild (5–9 points), moderate (10–14 points), or severe (15 or more points) [39]. For additional analyses, the categorical anxiety scores were further collapsed into binary anxiety variables (“no anxiety” and “anxiety”), where “anxiety” included mild, moderate, and severe anxiety scores.

### 2.3. Procedure

We recruited participants using convenience sampling through the authors’ personal social media accounts (Twitter and Facebook) and a national Master Gardeners email listserv. The authors’ original tweets were retweeted by institutional accounts and other Twitter users. The survey was developed, distributed, and stored on the online survey platform Qualtrics (Qualtrics, Provo, UT, USA) from 15 June 2020 through 23 September 2020. The online survey was preceded by an informed consent agreement.

### 2.4. Study Participants

A total of 1134 individuals completed the RANG survey from 15 June 2020 through 23 September 2020. Only individuals who were 18 years or older could complete the survey. Individuals were included in the study if they consented to participate and resided within the United States. A total of 1013 participants met the inclusion criteria and were included in the analyses (Table 1). Most participants were female (86%), white (82%), and not Hispanic or Latino (87%). The majority of participants were between the ages of 50 and 69 years (43%) and had either a graduate or professional degree (50%). Participants resided in 35 of 50 U.S. states, including Washington, D.C. The three most common states of residence were Maryland (50%), Texas (17%), and South Carolina (15%). Ninety-eight percent of participants were gardening at the time of the survey. Ninety-three percent of participants had not experienced COVID-19 symptoms, and 89% did not have a family member who had experienced COVID-19 symptoms (Table 1). However, approximately half (47%) of all participants had underlying health issues that made them more susceptible to COVID-19. This could be related to the large percentage of participants over 50 years old.

### 2.5. Statistical Analysis

Data downloaded from Qualtrics were cleaned of incomplete responses. Nonresponses to question 1, “Would you like to participate in this study?” or responses of “no” were removed. Nonresponses to question 2 (“Do you currently garden?”) and two participants who did not reside in the U.S. were also removed. Participants that selected American Indian/Alaskan Native, Native Hawaiian/Other Pacific, and any combination of two or more responses for race were grouped together into the category “Other” because of the low response rates in these categories. State of residence was reported for the three states with the most responses (Maryland 50%, South Carolina 15%, Texas 19%), and all other responses (18%) were collapsed into “Other” due to the low frequency of responses from other states.

Descriptive statistics, such as gardening status, demographics, and outdoor activities, were calculated. Chi-square test statistics were used to compare the differences between participants with and without anxiety by demographics, as well as gardening and outdoor activity variables. Chi-square and Fisher’s exact test using the Bonferroni correction were used to test for differences in participants with and without anxiety between demographic groups. The subcategory with the largest percentage of total respondents was chosen as the reference group for the chi-square and Fisher’s exact tests using the Bonferroni correction (Table 1). Additionally, a chi-squared test was used to test for an association between age and gardening experience. Due to the non-normal distribution of the continuous anxiety score variable (Shapiro-Wilk test, *p* < 0.001), Kruskal–Wallis and Mann–Whitney U-tests were performed to determine the differences in the continuous anxiety score by the aforementioned variables. Dwass, Steel, and Critchlow–Fligner tests were used to identify which variable categories had significant differences in anxiety scores. Logistic regression with a coding effect parameterization and Fisher’s scoring optimization technique were used to calculate the odds ratios of experiencing anxiety based on participant demographics, gardening and outdoor activities, and personal experience with COVID-19. The final set of predictors was determined by reviewing the existing literature and assessing the correlations between variables. In all cases, *p* -values of ≤0.05 were defined as statistically significant. All statistical analyses were performed using SAS System Software (SAS System Version 9.2, Cary, NC, USA).

## 3. Results

### 3.1. Anxiety

Forty-six percent of RANG participants reported some level of anxiety (Table 1). Anxiety prevalence differed significantly by the following demographic and COVID-19 related variables: age (*p* < 0.001), sex (*p* = 0.002), education (*p* = 0.04), state of residence (*p* < 0.001), having a family member who experienced COVID-19 symptoms (*p* = 0.01), and having underlying health issues related to COVID-19 susceptibility (*p* = 0.03) (Table 1). There were no significant differences in anxiety prevalence by race, ethnicity, or having personally experienced COVID-19 symptoms (Table 1).

Eighteen- to 29-year-olds and 30–49-year-olds had higher anxiety scores than 50–69- and 70–89-year-olds (*p* < 0.001) (Table 2). Similarly, 50–69-year-olds had higher anxiety scores than 70–89-year-olds (*p* < 0.001). Females had higher median anxiety scores compared to males (*p* < 0.001) (Table 2). Median anxiety scores differed significantly between the COVID-19 related variables by personal experience with COVID-19 symptoms (*p* = 0.03), having family members that experienced COVID-19 symptoms (*p* = 0.007), and having underlying health issues that increased COVID-19 susceptibility (*p* = 0.007) (Table 2).

### 3.2. Gardening

**Figure 1:**
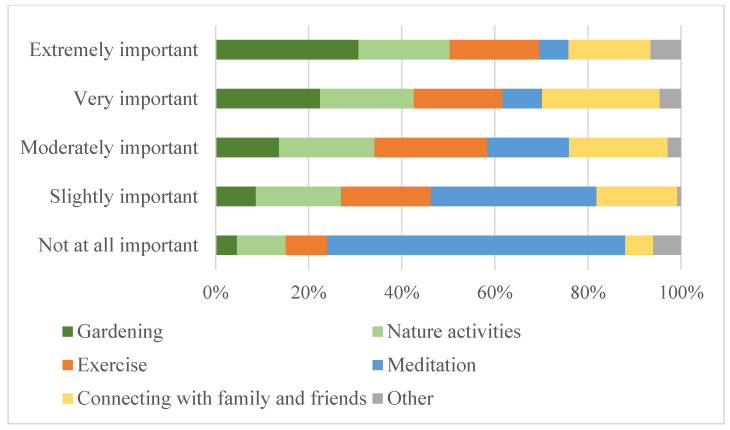
RANG participant ranking of stress management activities by level of importance.

Nearly all participants were gardening at the time of the survey (98%). Over half had been gardening for 15 or more years (60%), while 9% had been gardening for one year or less (Table 1). At the time of the survey, 85% of participants had spent at least three hours gardening over the past two weeks, and 49% had spent more than eight hours gardening (Table 1). Eighty-two percent of participants reported spending more time in their home garden during the study period than in the previous year, and 58% of participants had changed their gardening practices since the COVID-19 pandemic began (Table 1). For questions asking about motivation to start gardening and garden type, multiple responses could be selected. Participants’ top motivations to start gardening were to grow their own food (66%), for landscaping (62%), and for stress reduction (57%) (Table 1). The most popular types of gardens were flower (81%), vegetable (71%), container (66%), and in-ground (65%) gardens. RANG participants chose gardening and nature activities as the two most “extremely important” stress management activities during the pandemic (Figure 1).

### 3.3. Outdoor Activities

**Figure 2:**
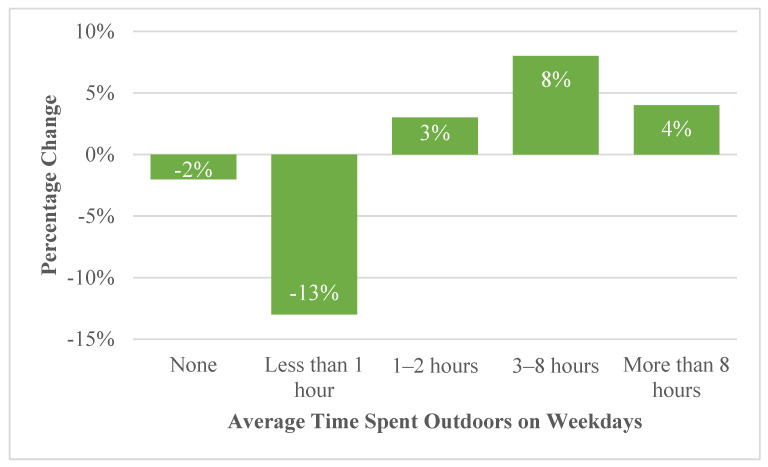
Percentage of RANG survey respondents reporting changes in amount of time spent outdoors on the weekdays during the summer of 2020 compared to the summer of 2019.

Most RANG participants spent 1–2 h (38%) or 3–8 h (35%) outside on weekdays and 3–8 h (52%) on weekends (Table 1). Participants reported spending more time outdoors on weekdays and weekends during the survey period compared to the previous year (Figure 2). The highest increase in time spent outside on weekdays was in the 3–8 h (8% increase) category (*p* < 0.001) (Figure 2). The largest change, however, was the 13% decrease in participants who spent less than one hour outside on weekdays (Figure 2). In addition to gardening, the most reported other outdoor activities over the two weeks preceding survey completion were “walking, hiking, backpacking, or camping” (73%), followed by “outdoor viewing, photography, and identification of vegetation” (68%), and “outdoor nature viewing, photography and identification of animal wildlife” (66%) (Table 1).

### 3.4. Impact of Gardening on Anxiety

Anxiety prevalence differed significantly by length of gardening experience and time spent gardening in the two weeks preceding the survey (*p* < 0.001; *p* = 0.04) (Table 1). In addition, fewer RANG participants who spent more time gardening due to the pandemic (*p* = 0.01) and whose motivation to start gardening was exercise (*p* < 0.001) had anxiety (Table 1). Interestingly, more RANG participants who reported changing their gardening practices due to the pandemic had anxiety compared with those who did not change their gardening practices (*p* < 0.001) (Table 1).

Participants who gardened had lower mean (5.1) and median (4) anxiety scores than non-gardeners (6.04; 6), but these findings were only marginally significant for mean scores (*p* = 0.06) and not significant for median scores (Table 1). Additionally, it is worth noting that the majority of participants (98%) were gardening at the time they completed the survey. Participants who had gardened for 15 or more years had lower median anxiety scores than those who had been gardening for 0–3 months (*p* < 0.001), 4–12 months (*p* = 0.03), 13 months–5 years (*p* < 0.001), and 6–10 years (*p* = 0.013) (Table 2). Notably, there was a significant association between age and gardening experience (*p* < 0.001); 50–69 and 70–89-year-olds accounted for 51% of individuals with 15 or more years of gardening experience. Participants who spent more than 8 h gardening had significantly lower median anxiety scores than those who spent 1–2 h gardening (*p* = 0.003) (Table 2).

### 3.5. Impact of Other Outdoor Activities on Anxiety

There were significant differences in anxiety presence/prevalence by amount of time spent outdoors on weekdays, but not on weekends (*p* < 0.001; Table 1). More participants who had been picnicking in the two weeks prior to the survey had anxiety (*p* < 0.001), but there were no significant differences in anxiety presence/prevalence for any other outdoor activities (Table 1).

Median anxiety scores decreased as time spent outdoors on weekdays increased (*p* < 0.001) (Table 2). Participants who spent less than one hour outdoors per weekday had significantly higher median anxiety scores than those who spent 1–2 h (*p* < 0.001), 3–8 h (*p* < 0.001), or more than 8 h (*p* < 0.001) outdoors per weekday (Table 2). Anxiety scores did not differ significantly by time spent outdoors on the weekend (Table 2). Considering specific outdoor activities, individuals who had engaged in water sports had lower anxiety scores (*p* = 0.05) than those who did not, and those who picnicked had higher anxiety scores (*p* < 0.001) than those who did not (Table 2). There were no significant differences in anxiety scores for any other outdoor activities.

### 3.6. Factors Impacting Anxiety

We identified six significant risk factors for having self-reported anxiety with our logistic regression model after adjusting for covariates (Table 3). The 50–69-year-olds and 70–89-year-olds had lower odds of self-reporting anxiety compared to 18–29-year-olds (OR = 0.34; 95% CI: 0.15, 0.77; OR = 0.21; 95% CI: 0.08, 0.53); males had lower odds of self-reporting anxiety than females (OR = 0.59; 95% CI: 0.35, 0.99); and Texas residents had lower odds of self-reporting anxiety than Maryland residents (OR = 0.49; 95% CI 0.31, 0.8). Participants who had picnicked over the past two weeks (OR = 1.79; 95% CI: 1.19, 2.71) and those who had changed their gardening practices due to the pandemic (OR = 1.73; 95% CI: 1.23, 2.43) had higher odds of having anxiety.

## 4. Discussion

Our study sought to assess anxiety among U.S. adults during the early stages of the COVID-19 pandemic using a cross-sectional online survey and evaluate the impact of gardening and other outdoor activities on anxiety. We documented high rates of self-reported anxiety among RANG participants with significant differences by age, sex, education, state, and COVID-19 related variables. RANG participants who had more gardening experience and spent more time in the garden had lower anxiety scores. In addition, we found that younger adults (18–29 years old) and women had higher odds of anxiety as well as those who reported changes in their gardening activities since the pandemic began.

### 4.1. Increased Anxiety Rates

Forty-six percent of RANG participants reported experiencing anxiety symptoms during the summer of 2020. Our anxiety findings are comparable to other survey-based studies during the same timeframe and are significantly higher than anxiety reports before the COVID-19 pandemic [9,44]. A survey by Czeisler et al., of over 5000 U.S. adults in June 2020 found that 31% reported anxiety or depression symptoms [9]. Despite most young adults being at low risk of experiencing physical health complications from a COVID-19 infection, the RANG study reflected U.S. and international trends showing that 18–29-year-olds were more likely to report anxiety symptoms based on our logistic regression analysis [9,45]. Seventy-five percent of 18–24-year-old respondents from the Czeisler et al. survey reported anxiety and depression symptoms, significantly more than any other age group surveyed [9]. Similarly, Stanton et al. [45] found that 18–45-year-old Australians had significantly higher depression, anxiety, and stress scores compared with older individuals in April 2020. The high percentage of young adults reporting anxiety may be a result of the indirect consequences of the pandemic, such as social isolation and economic instability [8,46,47]. The CDC has recognized concerns among young adults during the pandemic including changes in routines, challenges with employment and education, missed life events, and lack of continuity in health care [48]. One of our participants wrote in on the survey, “My 11-year-old daughter has found gardening an outlet for her during COVID. Never did we think our steady, easy-going daughter would struggle with anxiety because of this pandemic. But Jane [name changed for privacy] was having deep struggles. Jane and I now go out every morning and evening to tend our garden and talk about things. It’s been a lifeline for her to focus on beauty and life, truly.” The differing responsibilities, routines, work or retirement statuses, and societal expectations of the age groups could explain why, despite being at greater risk of adverse health outcomes, older populations have reported lower levels of anxiety when compared to young adults. Corley et al. examined mental and physical wellbeing in 84-year-olds during the COVID-19 lockdown in Scotland and found low levels of anxiety, with 64% reporting never feeling anxious about COVID-19 in the two weeks preceding the survey [32]. These findings are similar to the 73% of RANG participants aged 70–89 years old reporting no anxiety during the two weeks preceding the survey. While young adults may be more adaptive to a virtual format, older adults have more experience with an isolated routine. Additionally, the activities young adults must transition to online are rooted in social interactions, such as classroom learning, graduations, and physical contact, such as athletics and performance arts [47].

Females responding to the RANG survey had higher median anxiety scores and higher odds of anxiety than males based on our logistic regression model. Our findings are consistent with similar studies that have identified the female gender as a risk factor for anxiety, particularly during pandemics [49,50,51,52]. Previous studies have found that women are more susceptible than men to anxiety in general, and anxiety disorders are more prevalent in women [52,53,54]. The observed differences between women and men have been hypothesized to be the result of genetic or social factors, including learned coping behaviors, or a combination of the two [55]. During the H1N1 influenza crisis in Korea, another pandemic, a study by Kim et al., found that women had higher anxiety [49]. Ozdin and Ozdin discovered that women in Turkey experienced higher levels of both depression and anxiety than men during the first months of the COVID-19 pandemic [51]. The increased anxiety rates among women during the first year of the COVID-19 pandemic observed by the RANG survey and other studies could be related to the stress of increased childcare and other caregiving responsibilities, as women are disproportionately in caregiving roles globally and in the U.S. [56].

Ethnicity and education have also been identified as important predictor factors for anxiety during the COVID-19 pandemic. Czeisler et al., found that anxiety and depressive disorder symptoms were reported highest among Hispanic participants compared to non-Hispanic white and non-Hispanic Asian participants [9]. However, there were no differences in anxiety presence/prevalence by ethnicity among RANG participants. The lack of significant difference in anxiety by ethnicity in the RANG study could be attributed to the small percentage of Hispanic participants (3%). Furthermore, the deterioration of mental health among African Americans throughout the COVID-19 pandemic has been well documented. Due to systemic racism, African Americans have been more vulnerable to contracting COVID-19 and, as a result, have experienced disparate COVID-19 morbidity and mortality rates [57]. A greater proportion of African Americans has endured anxiety and other adverse mental health outcomes [58]. Since 2019, anxiety and depression symptoms have increased more than threefold among Americans, and African Americans have carried the largest burden [59]. Additionally, African Americans also experienced a spike in anxiety and depression as a result of the Racial Reckoning Summer of 2020 [60]. However, our results did not reveal these findings, most likely due to the small percentage of African American participants. Czeisler et al., also determined that educational attainment was inversely related to anxiety disorder [9]. In the RANG study, although anxiety presence/prevalence differed by education, there were no significant differences in anxiety score by education. This could be partly due to the disproportionate percentage of RANG participants that had earned at least a bachelor’s degree (80%).

State of residence also influenced anxiety levels among RANG participants, with those living in Texas having significantly lower odds of anxiety than participants living in Maryland. Vahratian et al. hypothesized that trends in anxiety could be correlated with COVID-19 case counts [10]. Maryland’s seven-day average of COVID-19 cases per 100,000 were 16, 6.7, and 14.8 on 1 June 2020, 6 July 2020, and 2 August 2020, respectively, compared to 4.4, 23.4, and 27.1 in Texas on those same dates [1,61]. The differences in anxiety between Texas and Maryland participants could instead be due to restrictions on travel and activities. In the U.S., each state government determined their own stay-at-home orders and related COVID-19 restrictions, such as travel. In Maryland, the stay-at-home order resulted in the closure of public spaces such as playgrounds and recreation fields/courts [6]. In contrast, the same order in Texas was not presented as a ‘stay-at-home’ order, which could have led to more Texans than Marylanders engaging in their usual activities and consequently experiencing less anxiety related to these changes [6,62].

### 4.2. Gardening and Health

Increases in gardening supply and seed sales across the U.S. during the early stages of the COVID-19 pandemic suggest that gardening increased beyond typical growth rates during this time on a national level. An analysis by Breck’s mail order gardening company found an 8.6% increase in monthly building and garden retail sales between Spring 2019 and Spring 2020 [63]. Increases in gardening could partly be explained by increased time spent at home [64]. Eighty-two percent of RANG participants reported increased gardening in 2020. Similarly, Corley et al. found that 50% of their participants had increased garden usage when compared to pre-lockdown, with 67% reported using gardening for relaxation [32]. When asked how their gardening practices changed during the pandemic, several participants in the RANG study noted in open-ended responses that working from home and the projected lack of summer vacations increased their capacity to care for a garden. One participant wrote “I’m transplanting and creating more container gardens since I do not anticipate traveling this summer knowing I will be available to water”. Another participant wrote, “Spent more time outside in my yard because I had more time since it was a safe place to be... found that it was great therapy during this crazy time we find ourselves in.” Interestingly, our logistic regression model found that participants who reported changes in gardening practices since the pandemic began had an increased risk of anxiety compared to those who did not change gardening practices (*p* = 0.002). This finding, combined with some of the open-ended responses, suggests that individuals experiencing anxiety might have turned to gardening as a coping mechanism. Gardening has previously been shown to be an effective mechanism for coping, reducing stress, and increasing resiliency [65]. Due to the cross-sectional design of our study, we are not able to determine the temporal relationship between anxiety and changes in gardening practices to know which came first for RANG participants.

The effectiveness of gardening as a mental health intervention has been explored by previous, and now emerging, studies amid the COVID-19 pandemic to determine whether positive effects, such as mood improvement, reduced anxiety, and trauma recovery are observed [23,30,66,67]. Participants of a study by Sunga and Advincula reported that their motivation to garden was to reduce stress, anxiety, and boredom brought on by the pandemic and that gardening improved their mood and behavior [66]. Mullins et al. reported that 71% of long-time and 62% of new gardeners used gardening for relaxation [67]. This theme is reflected in the RANG study with participants identifying gardening (44%) and nature activities (29%) as the most ‘extremely important’ stress management activities. One participant who was struggling with their mental health at the time of the survey noted in an open-ended response, “…I’ve been doing breathing exercises when I walk on the trail. If I didn’t have access to nature, I swear to God, I’d have killed myself by now”. Another participant discussed the mindfulness benefits of working in the garden, writing “Gardening is what I do to relieve stress. You can’t worry when you are engrossed in weeding, building rock walls, and installing stone paths, or planning and planting a garden. Having your hands in the earth connects you directly to nature, which is a welcome companion in my life. Nature is constant and beautiful”.

Home gardens can also be an effective strategy to enhance household food security and nutrition [68]. The pandemic exacerbated threats to food security through food chain disruptions and economic instability [69]. Food stockpiling and hoarding was seen throughout the U.S. and supply chains were further weakened by COVID-19 outbreaks in food processing facilities [70]. One RANG participant explained in an open-ended response how their increased gardening was directly tied to food shortages, saying “There were times when we couldn’t get certain fresh produce items like lettuce, fresh greens, and other food items at the grocery store because the shelves were empty. So, I ramped up the garden instead” Sixty-four percent of RANG participants reported growing food as motivation to start gardening, of which less than half (44%) reported experiencing anxiety. This may suggest that food gardeners felt less at risk of experiencing food insecurity because of their gardens/skills.

Almost all RANG participants were gardeners (98%), but with varying amounts of experience and time spent gardening. Although a lower percentage of RANG gardeners had anxiety compared to non-gardeners, this finding was only marginally significant (*p* = 0.06). Further, because the majority of RANG participants were gardeners, the findings comparing gardeners to non-gardeners should be repeated with a more balanced sample. Corley et al. similarly found that gardening vs. non-gardening groups did not significantly differ in self-reported anxiety or mental, emotional, or physical health [32]. The lack of any significant difference in anxiety between RANG gardeners and non-gardeners during the COVID-19 pandemic could be related to the general increase in anxiety among the U.S. population, as well as the large percentage of gardeners in our study sample. However, the duration of gardening could impact observed health benefits, as previous studies have noted certain longer time periods are needed to observe the effects of mindful practices [71,72]. There were significantly lower anxiety scores among RANG participants who had gardened for 15 or more years. We found significant associations between age and gardening experience among the RANG participants. Older participants might have more time and opportunity to garden for longer periods of time than younger participants. Additionally, previous studies have demonstrated that mindful practices such as meditation, yoga, and exercise can reduce blood pressure, depression, and anxiety either on their own or in combination with medication but may require anywhere from 3 to 24 weeks of practice before significant reductions in anxiety and depression symptoms are observed [71,72]. Gonzalez and colleagues found that positive health impacts such as reduced depression severity, increased life satisfaction, and increased cognitive function were present three months after gardening, further suggesting persistent health benefits [31].

We also found that increased time spent gardening was associated with significantly lower anxiety scores. Corley et al. identified that a higher frequency of gardening during the COVID-19 pandemic was associated with self-reports of better physical, emotional, and mental health but not anxiety; however, this could be a factor of their study population being composed entirely of 84-year-olds and low levels of anxiety being reported overall [32]. Several recent studies have identified a dose–response relationship between time spent outside and improved mental health [26]. One RANG participant commented that their gardening “…has increased considerably and I find that I need more time in the garden to help me with stress related to current events”.

Exercise as a motivating factor for gardening also differed significantly between RANG participants with and without anxiety. Gardening involves differing levels of physical exertion depending on the specific type of gardening. Physical activity has repeatedly been shown to improve physical and mental health [72,73]. Additionally, exercise outdoors enhances those health benefits [24,27,28].

### 4.3. Health Benefits of Other Outdoor Activity

More time spent outdoors on weekdays was associated with lower anxiety levels for RANG participants. Mounting evidence suggests that contact with nature produces a wide range of health benefits, both psychological and physiological, including reduced anxiety and mental fatigue, increased feelings of relaxation and happiness, and decreased blood pressure and cortisol levels [17,18,19,20,21]. One participant wrote about the therapeutic benefits they experienced from spending time outdoors, writing “Spending time outdoors is therapeutic no matter what is going on in your life or the world, but especially now”. Additionally, spending time outdoors increases Vitamin D intake, which has been shown to improve mental health and musculoskeletal health and to reduce the risk for diseases such as type 2 diabetes and chronic illnesses [74].

Anxiety levels did not differ significantly by time spent outside on weekends. One possible explanation is that fewer participants had to work on the weekends, resulting in people with varying anxiety levels spending time outdoors at leisure. Interestingly, when we explored specific outdoor activities that predict the odds of having anxiety, there were no significant associations except for increased odds of having anxiety for those who had picnicked in the past two weeks. Due to the fact that picnics generally involve being near others with no masks, individuals picnicking might have more fears about contracting COVID-19 from, or spreading the virus to, family and friends. It has been noted in previous research that picnics can be anxiety-inducing events for people on the autism spectrum disorder because of the divergence from normal routine [75].

### 4.4. Gardening and Outdoor Education Opportunities

Cultivating outdoor practices, including gardening, can provide short- and long-term health benefits to people of all ages, sexes, races, and ethnicities regardless of physical location. During the COVID-19 pandemic, gardening and other nature-related activities were reported as the two most important stress management activities among the RANG study participants. Time spent outdoors significantly reduced anxiety, and those who spent the most time gardening or had gardened the longest had the lowest levels of anxiety. Providing accessible gardening education materials could increase participation in these anxiety-reducing activities. Gardening tutorials and guides are offered through Extension at land grant institutions including the Master Gardener training program. Given the enhanced anxiety reduction and physical benefits from long-term gardening practice, the training, support, and gardening opportunities offered through the Master Gardeners could bolster mental and physical health benefits. Additionally, creating opportunities for outdoor activities and encouraging outdoor activities on weekdays should be further explored as a preventative health measure.

### 4.5. Limitations

The cross-sectional design of our study did not allow us to determine the temporal sequence of anxiety symptoms and gardening or other outdoor activities, and thus we cannot assume a cause–effect relationship between the dependent and independent variables. A potential bias was introduced due to the self-report survey used in the study, as participants could have underestimated or overestimated their responses. Additionally, larger effect sizes for health benefits from gardening have been identified in studies using pre–post designs versus those comparing gardeners to non-gardeners, as was done in the RANG study [76]. Sharing our survey through the Master Gardener email listserv could have biased the results towards more individuals with gardening experience and interest; however, the survey was also shared widely to non-gardeners through the authors’ personal social media accounts. A strength of our study was that the GAD-7 survey questions have been previously validated. Since anxiety levels were self-reported on a subjective scale, the number of participants reporting anxiety symptoms could have been higher than if direct measures had been used. However, collecting anxiety symptoms by self-report on the online survey allowed us to collect responses during the COVID-19 research restrictions and reach a wider audience. There were several additional topics that we did not ask questions about in the survey that could have further shed light on associations between gardening and outdoor activities with anxiety, including whether the participant worked outside of the home, participants’ essential worker status, the number of children in the home, income, and income changes during with the COVID-19 pandemic. These important topics should be explored in further studies. Finally, the survey was only available in English and distributed within the U.S., thus the generalizability of our results is limited to an English-speaking U.S. population.

### 4.6. Implications

The RANG study findings can be expounded using a social determinants of health paradigm that acknowledges the presence of race-, ethnic-, and socioeconomic-based inequities [77]. Specifically, the determinants of (a) income; (b) occupational class; and (c) home ownership can offer a theoretical understanding of the increase in gardening reported by 82% of the RANG participants in 2020. First, RANG participants were not asked about their level of income, nor whether their income changed during the pandemic; however, it can be strongly theorized that these were middle-to-high-income participants, based on the fact that 80% possessed degrees of higher education, and 82% self-identified as ‘White’. This theoretical leap stems from an understanding that, within the United States, the median White family holds approximately 5–8 times more wealth in comparison to median Black and Hispanic families, respectively [78]. The average annual income in 2020 of graduate or professional degree holders was over $80,000, nearly two times the median income for those with a high school diploma or less [79]. Second, many, if not most, essential and frontline workers continued to work outside the home while many others had the privilege of working from home throughout the pandemic. Furthermore, differences in essential vs. nonessential workers fall on lines of educational attainment, gender, race, and ethnicity. For example, essential workers are more likely to be African American, Hispanic, and/or with less educational attainment [80]. Additionally, less educational attainment has been found to significantly affect anxiety symptoms by other studies conducted during the pandemic [51]. Moreover, a third of these essential workers have been designated as economically vulnerable, due to their household earnings being less than US $40,000 a year. Third, the gap between White and Black homeownership rates widened in 2020. In 2020, the homeownership rate for White and African Americans was 74.4% and 43.1%, respectively, a 31.3% difference compared to a difference of 29.7% in 2019 [81,82]. This final determinant of homeownership speaks to the likelihood of having a front or back yard and thereby increasing one’s opportunity for gardening. Collectively, an understanding of these determinants, as well as the inequities of these determinants (e.g., owning a home, working from home, and being financially solvent) brings a nuanced understanding to these research findings. The privilege of gardening was simply implied when a participant stated that she “spent more time outside in [her] yard because [she] had more time”.

## 5. Conclusions

Gardening is one of many outdoor activities that has been shown to reduce the symptoms of anxiety and improve mental health. The exact mechanisms that detail how gardening improves mental health are still being explored, but the RANG study sheds light on several important variables. RANG participants were gardening more during the summer of 2020 in the midst of the COVID-19 pandemic to reduce their stress at a time when anxiety was increasing throughout the U.S. Anxiety was highest among females and 18–29-year-olds participating in the RANG study. More experience with gardening, more time spent gardening, and more time spent outdoors on weekdays were all associated with lower anxiety levels. The importance of gardening for stress management and the increased odds of anxiety among those reporting changes to gardening practices during the COVID-19 pandemic suggests that individuals were already turning to gardening to reduce anxiety. Sustained gardening and outdoor activities targeted to high-risk groups could be important tools to reduce and further support those experiencing anxiety and could be used to minimize mental health burdens.

## Figures and Tables

**Table 1:** Characteristics of the RANG survey respondents by anxiety presence.

Variable	Anxiety, *n* (%)			*p*-Value ^a^
	Yes	No	Total	
Age (years)				<0.001
18–29	40 (70)	17 (30)	57 (6)	Reference ^b^
30–49	186 (65)	100 (35)	286 (28)	0.45
50–69	180 (41)	259 (59)	439 (43)	<0.001
70–89	56 (27)	154 (73)	210 (21)	<0.001
I prefer not to answer	4 (22)	14 (78)	18 (2)	<0.001
Sex				0.002
Male	38 (33)	78 (67)	116 (11)	
Female	419 (48)	451 (52)	870 (86)	
Race				0.2
Asian	13 (54)	11 (46)	24 (2)	0.43
Black/African American	10 (46)	12 (55)	22 (2)	0.96
White	382 (46)	448 (54)	830 (82)	Reference
Other	25 (61)	16 (39.02)	41 (4)	0.06
I prefer not to answer	36 (39)	56 (61)	92 (9)	0.05
Ethnicity				0.5
Hispanic or Latino	11 (39)	17 (61)	28 (3)	0.44
Not Hispanic or Latino	406 (47)	463 (53)	869 (87)	Reference
I prefer not to answer	44 (42)	62 (58.49)	106 (11)	0.31
Education				0.04
High-school graduate/GED	13 (52)	12 (48)	25 (2)	0.66
Some college or associate degree	66 (40)	100 (60)	166 (16)	0.09
Bachelor’s degree	145 (48.17)	156 (52)	301 (30)	0.84
Graduate or professional degree	241 (47)	267 (53)	508 (50)	Reference
I prefer not to answer	1 (9)	10 (91)	11 (1)	0.01
State of residence				<0.001
Maryland	273 (54)	237 (46)	510 (50)	Reference
Texas	49 (29)	120 (71)	169 (17)	<0.001
South Carolina	48 (32)	100 (68)	148 (15)	<0.001
Other	97 (52)	89 (48)	186 (18)	0.75
Personally experienced COVID-19 symptoms				0.06
Yes	41 (57)	31 (43)	72 (7)	
No	426 (45)	515 (55)	941 (93)	
Family member experienced COVID-19 symptoms				0.01
Yes	64 (57)	48 (48)	112 (11)	
No	403 (45)	497 (55)	900 (89)	
COVID-19 susceptibility due to underlying health issues				0.03
Yes	204 (43)	275 (57)	479 (47)	
No	263 (49)	270 (51)	533 (53)	
Gardening				0.06
Yes	452 (46)	538 (54)	990 (98)	
No	15 (65)	8 (35)	23 (2)	
Gardening experience				<0.001
0–3 months	35 (67)	17 (33)	52 (5)	<0.001
4–12 months	26 (59)	18 (41)	44 (4)	0.01
13 months–5 years	80 (60)	54 (40)	134 (14)	<0.001
6–10 years	53 (48)	58 (52)	111 (11)	0.1
11–15 years	22 (42)	30 (58)	52 (5)	0.69
15+ years	235 (40)	360 (61)	595 (60)	Reference
Time spent gardening				0.04
None	17 (63)	10 (37)	27 (3)	0.03
Less than 1 hour	9 (45)	11 (55)	20 (2)	0.75
1–2 h	64 (57)	49 (43)	113 (11)	0.003
3–8 h	173 (48)	187 (52)	360 (36)	0.06
More than 8 h	204 (41)	288 (59)	492 (49)	Reference
Motivation to garden ^c^				
Grow my own food	290 (44)	362 (56)	652 (66)	0.2
Exercise	145 (39)	227 (61)	372 (38)	<0.001
Stress reduction	258 (46)	305 (54)	563 (57)	0.8
Landscaping	269 (44)	346 (56)	615 (62)	0.06
Family members	238 (46)	276 (54)	514 (52)	0.9
Other	89 (50)	88 (50)	177 (18)	0.2
Increased time spent gardening due to COVID-19				0.01
Yes	384 (48)	421 (52)	805 (82)	
No	67 (37)	112 (63)	179 (18)	
Gardening practices changed due to COVID-19				<0.001
Yes	305 (54)	265 (46)	570 (58)	
No	147 (35)	270 (65)	417 (42)	
Time spent outdoors per weekday				<0.001
None	2 (67)	1 (33)	3 (3)	0.51
Less than 1 h	72 (71)	30 (29)	102 (10)	<0.001
1–2 h	184 (48)	200 (52)	384 (38)	Reference
3–8 h	149 (42)	206 (58)	355 (35)	0.11
More than 8 h	59 (36)	106 (64)	165 (16)	0.009
Time spent outdoors per weekend				0.9
None	1 (50)	1 (50)	2 (0.1)	1 ^d^
Less than 1 hour	15 (52)	14 (48)	29 (3)	0.45
1–2 h	95 (48)	105 (53)	200 (20)	0.48
3–8 h	236 (45)	293 (55)	529 (52)	Reference
More than 8 h	120 (48)	132 (52)	252 (25)	0.43
Bicycling for fun, exercise, or commuting in the past 2 weeks				0.4
Yes	144 (44)	182 (56)	326 (32)	
No	323 (47)	363 (53)	686 (68)	
Picnicking in the past 2 weeks				<0.001
Yes	116 (59)	79 (41)	195 (19)	
No	351 (43)	466 (57)	817 (81)	
Outdoor walking, hiking, backpacking, or camping in the past 2 weeks				0.4
Yes	347 (47)	393 (53)	740 (73)	
No	119 (44)	152 (56)	271 (27)	
Outdoor nature viewing, photography, or identification of animal wildlife in the past 2 weeks				0.9
Yes	309 (46)	358 (54)	667 (66)	
No	158 (46)	187 (54)	345 (34)	
Outdoor nature viewing, photography, or identification of vegetation in the past 2 weeks				0.5
Yes	323 (47)	366 (53)	689 (68)	
No	144 (44)	180 (56)	324 (32)	
Water sports in the past 2 weeks				0.3
Yes	55 (42)	77 (58)	132 (13)	
No	411 (47)	468 (53)	879 (87)	
Total	467 (46)	546 (54)	1013	

^a^ The *p*-values represent differences between participants with and without anxiety symptoms. ^b^ The reference group is the group against which the other subcategories were compared to determine if there were significant differences in anxiety between subcategories. ^c^ Participants could choose multiple responses to the survey question about motivation to garden. ^d^ This *p*-value was calculated using Fisher’s exact test.

**Table 2:** Kruskal–Wallis and Mann–Whitney U-test analyses of median anxiety scores by demographic, gardening, and outdoor activity variables.

Variable	Mean	Median	Interquartile Range	*p*-Value ^a^
Age				<0.001
18–29	7.39	7.00	7.00	
30–49	7.06	6.00	7.00	
50–69	4.66	3.00	6.00	
70–89	2.87	1.50	5.00	
Sex				<0.001
Female	5.32	4.00	6.00	
Male	3.53	3.00	5.00	
Education				0.54
Bachelor’s Degree	5.41	4.00	7.00	
Graduate or professional degree	5.12	4.00	5.00	
High-school graduate/GED	4.80	5.00	6.00	
Some college or associate degree	4.73	3.00	7.00	
Personally experienced COVID-19 symptoms				0.03
Yes	5.96	5.00	6.00	
No	5.04	4.00	6.00	
Family member experienced COVID-19 symptoms				0.007
Yes	6.36	5.00	7.00	
No	4.95	4.00	6.00	
COVID-19 susceptibility due to underlying health issues				0.007
Yes	4.75	4.00	6.00	
No	5.44	4.00	6.00	
Gardening Experience				<0.001
0–3 months	7.81	7.00	8.50	
4–12 months	6.27	5.00	5.50	
13 months–5 years	6.66	6.00	7.00	
6–10 years	5.54	4.00	6.00	
11–15 years	5.19	4.00	4.00	
15+ years	4.31	3.00	5.00	
Time spent gardening				0.001
None	3.75	4.50	2.50	
Less than 1 h	4.00	4.00	8.00	
1–2 h	6.42	5.00	7.00	
3–8 h	5.41	4.00	6.00	
More than 8 h	4.51	3.00	6.00	
Time spent outdoors per weekday				<0.001
None	8.33	5.00	12.00	
Less than 1 h	7.40	6.00	7.00	
1–2 h	5.25	4.00	7.00	
3–8 h	4.65	4.00	6.00	
More than 8 h	4.37	3.00	6.00	
Time spent outdoors per weekend				0.2
None	4.50	4.50	1.00	
Less than 1 hour	6.28	5.00	7.00	
1–2 h	4.99	4.00	6.00	
3–8 h	4.85	4.00	6.00	
More than 8 h	5.61	4.00	6.00	
Picnicking in the past 2 weeks				<0.001
Yes	6.31	6.00	7.00	
No	4.82	4.00	6.00	
Water sports in the past 2 weeks				0.05
Yes	5.24	4.00	6.00	
No	4.24	4.00	5.00	

^a^ The *p*-value compares median anxiety scores by the listed demographic, gardening, or outdoor activity variable.

**Table 3:** Logistic regression models predicting anxiety using demographic characteristics, COVID-19 experiences, gardening, and outdoor activities.

Characteristic	Unadjusted Odds Ratio (95% CI)	Adjusted Odds Ratio ^a^ (95% CI)	*p*-Value for Adjusted OR
Age (ref = 18–29)			
30–49	0.79 (0.43, 1.47)	0.87 (0.4, 1.9)	0.73
50–69	0.3 (0.16, 0.54)	0.34 (0.15, 0.77)	0.01
70–89	0.16 (0.08, 0.29)	0.21 (0.08, 0.53)	0.001
I prefer not to answer	0.12 (0.04, 0.42)	0.28 (0.04, 1.85)	0.18
Sex (ref = Female)			
Male	0.53 (0.35, 0.79)	0.59 (0.35, 0.99)	0.05
I prefer not to answer	0.46 (0.18, 1.21)	0.53 (0.11, 2.64)	0.44
Other	3.22 (0.33, 31.07)	0.22 (0.01, 8.10)	0.41
Race (ref = White)			
Asian	1.39 (0.61, 3.13)	1.06 (0.36, 3.11)	0.91
Black/African American	0.98 (0.42, 2.29)	0.43 (0.14, 1.26)	0.12
I prefer not to answer	0.75 (0.49, 1.17)	0.65 (0.23, 1.83)	0.41
Other	1.83 (0.96, 3.48)	1.27 (0.52, 3.07)	0.60
Ethnicity (ref = not Hispanic or Latino)			
Hispanic or Latino	0.74 (0.34, 1.59)	0.53 (0.20, 1.45)	0.22
I prefer not to answer	0.81 (0.54, 1.22)	1.87 (0.72, 4.86)	0.20
Education (ref = High-school graduate/GED)			
Some college or associate degree	0.61 (0.26, 1.42)	0.76 (0.26, 2.19)	0.61
Bachelor’s degree	0.86 (0.38, 1.94)	0.75 (0.27, 2.11)	0.59
Graduate or professional degree	0.83 (0.37, 1.86)	0.81 (0.29, 2.27)	0.69
I prefer not to answer	0.09 (0.01, 0.83)	0.10 (0.01, 1.34)	0.20
State (ref = Maryland)			
South Carolina	0.42 (0.28, 0.61)	0.72 (0.45, 1.15)	0.17
Texas	0.35 (0.24, 0.52)	0.49 (0.31, 0.8)	0.004
Other	0.95 (0.68, 1.32)	0.89 (0.58, 1.38)	0.61
Time gardening (ref = none)			
Less than 1 h	0.82 (0.10, 7.02)	0.86 (0.05, 16.48)	0.92
1–2 h	1.31 (0.18, 9.60)	2.12 (0.13, 33.83)	0.60
3–8 h	0.93 (0.13, 6.64)	2.33 (0.15, 36.41)	0.55
More than 8 h	0.71 (0.10, 5.07)	1.96 (0.12, 30.70)	0.63
How long have you been gardening? (ref = 15+ years)			
0–3 months	3.15 (1.73, 5.76)	1.18 (0.51, 2.76)	0.70
4–12 months	2.21 (1.19, 4.13)	0.77 (0.33, 1.80)	0.55
13 months–5 years	2.27 (1.55, 3.33)	1.11 (0.64, 1.92)	0.72
6–10 years	1.40 (0.93, 2.10)	0.78 (0.44, 1.38)	0.39
11–15 years	1.12 (0.63, 2.00)	0.69 (0.34, 1.39)	0.30
Motivation to start gardening (ref = not selected) ^b^			
Grow my own food	1.02 (0.78, 1.33)	0.72 (0.51, 1.01)	0.06
Exercise	0.69 (0.51, 0.93)	1.03 (0.71, 1.50)	0.87
Stress reduction	1.75 (1.32, 2.32)	1.37 (0.97, 1.95)	0.08
Landscaping	0.70 (0.54, 0.92)	0.73 (0.52, 1.02)	0.07
Family members (parents, grandparents, etc.)	0.96 (0.74, 1.23)	1.30 (0.93, 1.80)	0.12
Outdoor activities (ref = no)			
Have you done any type of bicycling for fun, exercise, or commuting over the last 2 weeks?	0.89 (0.68, 1.16)	0.77 (0.54, 1.09)	0.14
Did you go picnicking over the last 2 weeks?	1.95 (1.42, 2.68)	1.79 (1.19, 2.71)	0.01
Have you done any type of outdoor walking, hiking, backpacking, or camping over the last 2 weeks?	1.13 (0.85, 1.49)	0.76 (0.53, 1.10)	0.15
Have you done any outdoor nature viewing, photography, or identification of animal wildlife over the last 2 weeks?	1.02 (0.79, 1.33)	0.90 (0.60, 1.35)	0.61
Have you done any outdoor nature viewing, photography, or identification of vegetation over the last 2 weeks?	1.10 (0.85, 1.44)	1.42 (0.93, 2.15)	0.10
Have you done any motor boating, water skiing, jet skiing, canoeing, kayaking, rafting, tubing, surfing, sailboarding, or any form of boating over the last 2 weeks?	0.81 (0.56, 1.18)	0.78 (0.49, 1.26)	0.31
Have you done any other nature-related activities over the last 2 weeks?	0.89 (0.67, 1.19)	0.80 (0.57, 1.12)	0.20
Time Outside: Weekdays (ref = none)			
Less than 1 h	1.20 (0.11, 13.73)	1.16 (0.03, 43.47)	0.94
1–2 h	0.46 (0.04, 5.12)	0.47 (0.01, 16.95)	0.68
3–8 h	0.36 (0.03, 4.03)	0.52 (0.01, 19.03)	0.72
More than 8 h	0.28 (0.03, 3.14)	0.42 (0.01, 15.51)	0.63
Time Outside Weekend (ref = none) ^c^			
Less than 1 h	1.07 (0.06, 18.82)		
1–2 h	0.91 (0.06, 14.67)		
3–8 h	0.81 (0.05, 12.95)		
More than 8 h	0.91 (0.06, 14.70)		
This time last year, how much time did you spend outdoors per weekday (ref = none)			
Less than 1 h	0.64 (0.25, 1.61)	1.14 (0.35, 3.74)	0.83
1–2 h	0.39 (0.15, 0.96)	1.12 (0.33, 3.72)	0.86
3–8 h	0.28 (0.11, 0.71)	1.16 (0.33, 4.06)	0.82
More than 8 h	0.19 (0.07, 0.50)	1.02 (0.26, 3.92)	0.98
COVID (ref = no)			
Experienced COVID symptoms yourself	1.60 (0.99, 2.60)	1.25 (0.63, 2.49)	0.53
Family member experienced COVID symptoms	1.64 (1.11, 2.45)	1.42 (0.82, 2.48)	0.21
COVID susceptibility	0.76, (0.59, 0.98)	1.11 (0.79, 1.56)	0.53
Have you spent more time in your home garden since the shelter-in-place orders?	1.53 (1.10, 2.13)	0.96 (0.61, 1.49)	0.85
Have your gardening practices changed since the COVID-19 pandemic began?	2.11 (1.63, 2.74)	1.73 (1.23, 2.43)	0.002

^a^ This model was adjusted for all covariates in the above table (age, sex, race, ethnicity, education, state of residence, time spent gardening, length of time gardening, motivation to start gardening, outdoor activities, time outdoors on weekdays, change in time gardening, and experience with COVID). ^b^ One logistic regression was used that included all options, since participants could select more than one response for these questions. ^c^ Time spent outdoors on weekends was not included in the model because of the high correlation with time spent outdoor on weekdays.

## Data Availability

The data presented in this study are available on request from the corresponding author. The data are not publicly available due to privacy restrictions.

## Supplementary Materials

The following supporting information can be downloaded at: https://www.mdpi.com/article/10.3390/ijerph19095121/s1, RANG Survey.
