# Supplementary material for: Reducing Anxiety with Nature and Gardening (RANG): Evaluating the Impacts of Gardening and Outdoor Activities on Anxiety among U.S. Adults during the COVID-19 Pandemic
Source: Int J Environ Res Public Health. 2022 Apr 22;19(9):5121. doi: 10.3390/ijerph19095121 (PMC9100102; doi:10.3390/ijerph19095121)
Supplement: Supplementary file 1 [file ijerph-19-05121-s001.zip › ijerph-1640005-supplementary.pdf]

International Journal of
Environmental Research and Public Health

MDPI

Supplementary materials:

RANG Survey

1. Would you like to participate in this study?
    a. Yes
    b. No

2. Do you currently garden?
    a. Yes
    b. No

3. What type of garden do you work in (select all that apply)?
    a. Vegetable garden
    b. Flower garden
    c. Container garden
    d. Raised beds
    e. In-ground garden
    f. Member of a public or community garden
    g. Other

4. How long have you been gardening?
    a. 0–3 months
    b. 4–12 months
    c. 13 months–5 years
    d. 6–10 years
    e. 11–15 years
    f. 15+ years

5. What motivated you to start gardening (select all that apply)?
    a. Grow my own food
    b. Exercise
    c. Stress reduction
    d. Landscaping
    e. Family members (parents, grandparents, etc.)
    f. Other

6. Are you a Master Gardener?
    a. Yes
    b. No
    c. I don’t know what a Master Gardener is

7. How long have you been a Master Gardener?
    a. 0–5 years
    b. 6–10 years
    c. 11–15 years
    d. 15+ years

8. How much time have you spent gardening over the last 2 weeks?
    a. None
    b. Less than 1 hour
    c. 1–2 hours
    d. 3–8 hours
    e. More than 8 hours

9. How much time have you spent outdoors per weekday over the last 2 weeks?
    a. None
    b. Less than 1 hour
    c. 1–2 hours
    d. 3–8 hours
    e. More than 8 hours

10. Last year at this time, how much time did you spend outdoors per weekday?
    a. None
    b. Less than 1 hour
    c. 1–2 hours
    d. 3–8 hours
    e. More than 8 hours

11. How much time have you spent outdoors per weekend over the last 2 weeks?
    a. None
    b. Less than 1 hour
    c. 1–2 hours
    d. 3–8 hours
    e. More than 8 hours

12. Last year at this time, how much time did you spend outdoors per weekend?
    a. None
    b. Less than 1 hour
    c. 1–2 hours

d. 3–8 hours

e. More than 8 hours

13. Have you done any type of bicycling for fun, exercise, or commuting over the last 2 weeks?

a. Yes

b. No

14. Did you go picnicking over the last 2 weeks?

a. Yes

b. No

15. Have you done any type of outdoor walking, hiking, backpacking, or camping over the last 2 weeks?

a. Yes

b. No

16. Have you done any outdoor nature viewing, photography, or identification of animal wildlife, (birds, wildlife, fish, deer, bears, snakes, butterflies, turtles, and so on) over the last 2 weeks?

a. Yes

b. No

17. Have you done any outdoor viewing, photography, or identification of vegetation (wildflowers, grasses, trees, etc.) over the last 2 weeks?

a. Yes

b. No

18. Have you gone sailing, motor boating, water skiing, jet skiing, canoeing, kayaking, rafting, tubing, surfing, sailboarding, or any other form of boating over the last 2 weeks?

a. Yes

b. No

19. Have you done any other outdoor or nature-related activities over the last 2 weeks? (If yes, please specify)

a. Yes (If you choose yes, please write down the specific outdoor or nature-related activities in the box below.)

b. No

20. Since the shelter-in-place orders for the COVID-19 (coronavirus) pandemic began, have you spent more time in your home garden than last year?

a. Yes

b. No

21. Have your gardening practices changed since the COVID-19 (coronavirus) pandemic began?
    a. Yes
    b. No

22. How have your gardening practices changed?

23. How important have each of the following activities been in helping you manage stress since the shelter-in-place requirements began?

	Not At All Important	Slightly Important	Moderately Important	Very Important	Extremely Important
Gardening	○	○	○	○	○
Nature activities	○	○	○	○	○
Exercise	○	○	○	○	○
Meditation	○	○	○	○	○
Connecting with friends and family	○	○	○	○	○
Other (please specify)	○	○	○	○	○

24. Have you experienced any COVID-19 symptoms (cough, shortness of breath or difficulty breathing, fever, chills, muscle pain, sore throat, new loss of taste or smell)?
    a. Yes
    b. No

25. Has anyone in your family experienced any COVID-19 symptoms?
    a. Yes
    b. No

26. Do you have any underlying health issues that make you more susceptible to COVID-19 (65 years or older, chronic lung disease, asthma, serious heart conditions, compromised immune system, severe obesity, diabetes, chronic kidney disease undergoing dialysis, or liver disease)?
    a. Yes
    b. No

27. Over the last 2 weeks, how often have you been bothered by the following problems?

	Not at all	Several days	More than half the days	Nearly every day
Feeling nervous, anxious or on edge	○	○	○	○
Not being able to stop or control worrying	○	○	○	○
Worrying too much about different things	○	○	○	○
Trouble relaxing	○	○	○	○
Being so restless that it's hard to sit still	○	○	○	○
Becoming easily annoyed or irritable	○	○	○	○
Feeling afraid, as if something awful might happen	○	○	○	○

28. Our project team will be developing educational events and materials to provide information and best practices for gardening as a stress management technique. How would you like to receive this information? (select all that apply):
    a. Online workshop or class
    b. Virtual one-on-one consultation
    c. In-person one-on-one consultation
    d. Written materials or guides
    e. Online videos
    f. Other

29. What information would be helpful regarding gardening? (Select all that apply)
    a. Basic gardening techniques
    b. Container gardening
    c. Vegetable gardening
    d. Indoor plants
    e. Pest management
    f. Edible gardening
    g. Gardening and health
    h. Other

30. Please select your state of residence

31. Please enter your zip code:

32. What is your ethnicity?
    a. Hispanic or Latino
    b. Not Hispanic or Latino
    c. I prefer not to answer

33. Which category best describes your race (check all that apply)?
    a. American Indian/Alaskan Native
    b. Asian (Includes India and Middle East)
    c. Black/African-American
    d. Native Hawaiian/Other Pacific
    e. White
    f. Other
    g. I prefer not to answer

34. What is your age?
    a. 18–29
    b. 30–49
    c. 50–69
    d. 70–89
    e. Over 90
    f. I prefer not to answer

35. What is your sex?
    a. Male
    b. Female
    c. Other
    d. I prefer not to answer

36. What is the highest level of education you have completed?
    a. Less then high school
    b. High-school graduate/GED
    c. Some college or associate's degree
    d. Bachelor's degree
    e. Graduate or professional degree
    f. I prefer not to answer

37. Is there anything else you would like us to know?
